# Matrix Metalloproteinase-10 Promotes *Kras-*Mediated Bronchio-Alveolar Stem Cell Expansion and Lung Cancer Formation

**DOI:** 10.1371/journal.pone.0026439

**Published:** 2011-10-17

**Authors:** Roderick P. Regala, Verline Justilien, Michael P. Walsh, Capella Weems, Andras Khoor, Nicole R. Murray, Alan P. Fields

**Affiliations:** 1 Department of Cancer Biology, Mayo Clinic College of Medicine, Jacksonville, Florida, United States of America; 2 Department of Pathology, Mayo Clinic College of Medicine, Jacksonville, Florida, United States of America; Technische Universität München, Germany

## Abstract

Matrix metalloproteinase 10 (MMP-10; stromelysin 2) is a member of a large family of structurally related matrix metalloproteinases, many of which have been implicated in tumor progression, invasion and metastasis. We recently identified *Mmp10* as a gene that is highly induced in tumor-initiating lung bronchioalveolar stem cells (BASCs) upon activation of oncogenic *Kras* in a mouse model of lung adenocarcinoma. However, the potential role of *Mmp10* in lung tumorigenesis has not been addressed. Here, we demonstrate that *Mmp10* is overexpressed in lung tumors induced by either the smoke carcinogen urethane or oncogenic *Kras.* In addition, we report a significant reduction in lung tumor number and size after urethane exposure or genetic activation of oncogenic *Kras* in *Mmp10* null (*Mmp10^−/−^*) mice. This inhibitory effect is reflected in a defect in the ability of *Mmp10*-deficient BASCs to expand and undergo transformation in response to urethane or oncogenic *Kras in vivo* and *in vitro*, demonstrating a role for *Mmp10* in the tumor-initiating activity of *Kras*-transformed lung stem cells. To determine the potential relevance of MMP10 in human cancer we analyzed Mmp10 expression in publicly-available gene expression profiles of human cancers. Our analysis reveals that MMP10 is highly overexpressed in human lung tumors. Gene set enhancement analysis (GSEA) demonstrates that elevated *MMP10* expression correlates with both cancer stem cell and tumor metastasis genomic signatures in human lung cancer. Finally, Mmp10 is elevated in many human tumor types suggesting a widespread role for Mmp10 in human malignancy. We conclude that *Mmp10* plays an important role in lung tumor initiation via maintenance of a highly tumorigenic, cancer-initiating, stem-like cell population, and that Mmp10 expression is associated with stem-like, highly metastatic genotypes in human lung cancers. These results indicate that Mmp10 may represent a novel therapeutic approach to target lung cancer stem cells.

## Introduction

Non-small cell lung cancer (NSCLC) is the most common cause of cancer death in the United States [1]. Despite advances in treatment, clinical outcome of lung cancer patients remains poor. Therefore, there continues to be a need to identify underlying mechanisms of lung tumorigenesis that could lead to more effective means of prevention, diagnosis, prognosis and targeted therapies.

Emerging evidence supports the existence of rare subpopulations of cancer cells with stem-like characteristics [2], [3], [4]. These cancer-initiating cells or cancer stem cells (CSCs) exhibit self-renewal, tumor-initiating activity, and the ability to support tumor maintenance and metastasis [2], [4], [5], [6]. Thus, CSCs appear to be critical targets for effective, potentially curative cancer treatment. Unfortunately, CSCs exhibit intrinsic resistance to chemotherapy [7], [8], underlining the need to identify new therapeutic targets to effectively eradicate CSCs. CSCs share molecular and genomic features with embryonic stem cells, and embryonic stem cell genomic signatures are enriched in highly tumorigenic cancer stem cells. Such CSCs have been described in leukemia [9], and solid tumors, including melanoma [10], breast [11], brain [12], [13], [14], prostate [15], head and neck [16], pancreatic [17], colon carcinomas [18], [19], and lung [20], [21].

We recently demonstrated that Mmp10 is required for the transformed growth and invasion of human NSCLC cells *in vitro* [22]. However, the role of Mmp10 in lung tumorigenesis has not been addressed. Here, we use a combination of mouse carcinogenesis models and analysis of human tumors to demonstrate that Mmp10 plays a novel, unexpected role in *Kras*-mediated lung cancer initiation, lung cancer stem cell expansion, and metastasis. Our data indicate that Mmp10 is an attractive therapeutic target for CSCs.

## Results

### Mmp10 loss inhibits Kras-mediated lung tumorigenesis

**Figure 1:**
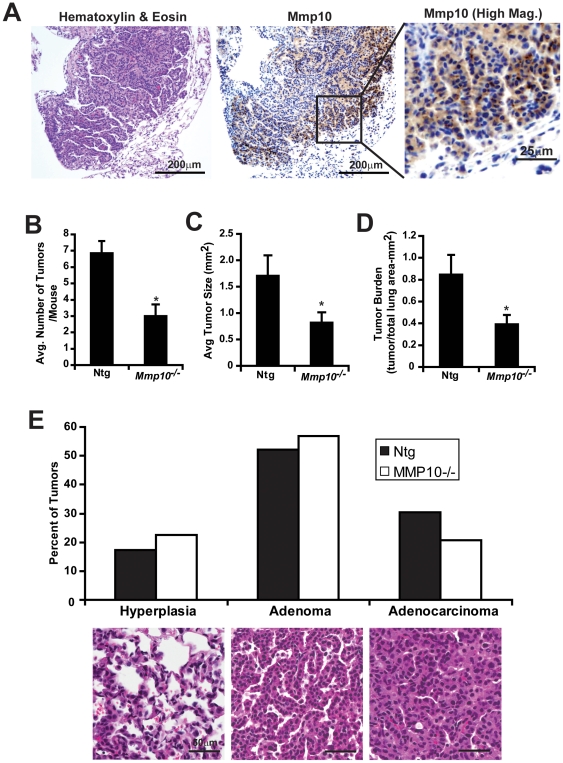
Mmp10 plays a promotive role in urethane-induced lung tumorigenesis. *Mmp10^−/−^* mice and Ntg littermates were injected with urethane and analyzed as described in *Materials and Methods*. **A**) H & E and immunohistochemical staining for Mmp10 in urethane-induced lung tumors. Higher magnification image of Mmp10 immunostaining is shown in the inset. Quantitative analysis of tumor number **B**), tumor size **C**) and tumor burden **D**) in urethane-treated Ntg (n = 7) and *Mmp10^−/−^* (n = 12) mice. Mean +/−SEM; p<. 0.012 tumor number; p<0.019 tumor burden; p = 0.034 tumor size). **E**) Urethane-induced tumors from Ntg and Mmp10*^−^* ^/*−*^ mice were graded as hyperplasia, adenoma or adenocarcinoma using published criteria [26]. Results are presented as the percentage of total tumors of each grade. Statistical analysis using Mann-Whitney U test revealed no statistically significant difference in tumor grade between urethane-induced tumors in Ntg and Mmp10*^−^* ^/*−*^ mice (p = 0.39).

In order to determine whether *Mmp10* is involved in lung tumor formation, mice were treated with the smoke carcinogen urethane to induce lung adenocarcinoma tumors using well-established protocols [23]. Immunohistochemical analysis demonstrated that Mmp10 expression is elevated in urethane-induced tumors, particularly at areas of contact between the tumor and the surrounding stroma (**Figure 1A**). These results are consistent with previous observations in human NSCLC tumors [22], [24], [25]. Interestingly, when Mmp10*^−^* ^/*−*^ mice were exposed to urethane, these mice developed significantly fewer (**Figure 1B**) and smaller (**Figure 1C**) tumors, and exhibited a smaller total tumor burden (**Figure 1D**) than non-transgenic (NTg) littermates. Analysis of tumor grade using the system described by Kelly-Spratt et al. [26] demonstrated that urethane-induced tumors from Ntg and Mmp10*^−^* ^/*−*^ mice showed a similar distribution of tumors along the hyperplasia-adenoma-carcinoma progression scheme (**Figure 1E**). These data indicate that Mmp10 plays an important promotive role in urethane-induced lung tumorigenesis primarily at the tumor initiation stage.

**Figure 2:**
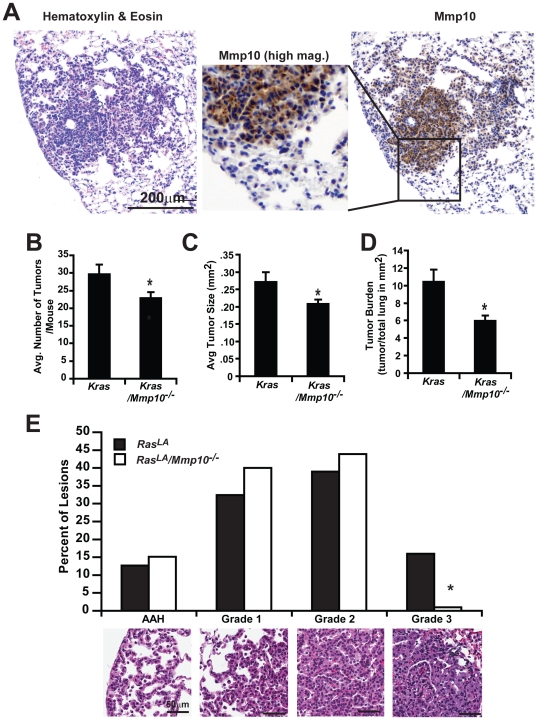
Mmp10 is necessary for *Kras^LA2^*-induced lung tumorigenesis *in vivo.* **A**) Immunohistochemical staining of *Kras^LA2^* lung tumor for mouse MMP10. Higher magnification image of Mmp10 immunostaining is shown in the inset. Quantitative analysis of **B**) tumor number, **C**) tumor size and **D)** tumor burden in *Kras^LA2^* and *Kras^LA2^/Mmp10^−/−^* mice. Columns, mean; bars, SEM, n = 13, (*) denotes p = 0.04. E) Tumors from *Kras^LA2^* and *Kras^LA2^/Mmp10^−/−^* mice were categorized as advanced adenomatous hyperplasia (AAH), or grade 1,2 or 3 adenomas using the published scoring criteria described by Jackson et al. [28]. Results are presented as the percentage of total tumors of each grade. Statistical analysis using Mann-Whitney U test revealed a significant decrease in higher grade tumors in *Kras^LA2^/Mmp10^−/−^* mice; *p<0.002.

Since urethane-induced lung tumorigenesis is thought to be driven, at least in part, through acquisition of *Kras* mutations, we assessed whether Mmp10 plays a similar promotive role in *Kras*-mediated lung tumorigenesis. For this purpose, we crossed Mmp10*^−^* ^/*−*^ mice to *Kras^LA^* mice, in which spontaneous recombination events lead to activation of a mutant *Kras^G12D^* allele in the lung that drives lung tumor formation [27]. Lung tumors in *Kras^LA^* mice express elevated Mmp10 that exhibits a similar pattern of expression as in urethane-induced lung tumors (**Figure 2A**). Similar to our observation in urethane-treated mice, bitransgenic *Kras^LA2^*/*Mmp10^−/−^* mice developed fewer (**Figure 2B**) and smaller (**Figure 2C**) tumors, and exhibit lower overall tumor burden (**Figure 2D**) than *Kras^LA2^* mice. Analysis of tumor grade using the scoring system devised by Jackson et al. [28] demonstrated that ∼16% of tumors in *Kras^LA^* mice were high grade adenocaricnomas (grade 3), whereas less than 1% of the tumors in *Kras^LA2^/Mmp10^−/−^* mice were grade 3 (**Figure 2E**). These data suggest that Mmp10 in this model is important in both tumor initiation and progression. Taken, together our data indicate that *Mmp10* plays an important role in multiple models of lung tumor formation.

### Mmp10 is required for Kras-mediated bronchio-alveolar stem cell expansion in vivo

**Figure 3:**
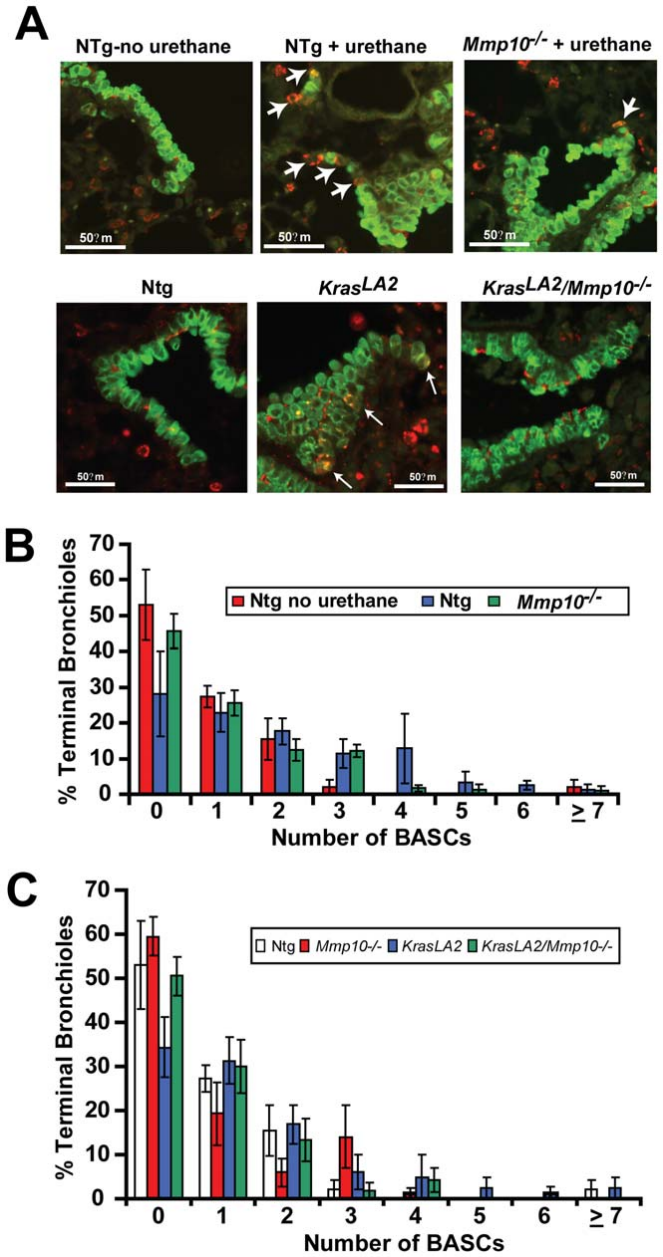
MMP10 is required for urethane- and *Kras-*induced BASC expansion *in vivo*. **A**) Immunofluorescent analysis of CCSP (green) and SPC (red) dual positive BASCs (white arrows) in terminal bronchioles (TB) of control Ntg, and urethane-treated Ntg and *Mmp10^−/−^*mice (***upper panels***), and from Ntg, *Kras^LA2^* and *Kras^LA2^/Mmp10^−/−^* mice (***lower panels***). **B**) Quantitative analysis of BASCs in control Ntg, and urethane treated Ntg and *Mmp10^−/−^* mice. %TBs; bars +/−SEM, n = ≥30 TBs/genotype. p<0.0005 urethane treated NTg vs. *Mmp10^−/−^*; p<0.0001 untreated Ntg vs. urethane-treated Ntg; No significant difference between untreated non-Ntg vs. urethane treated *Mmp10^−/−^* mice. **C**) Quantitative analysis of BASCs in lung TBs of Ntg, *Kras^LA2^* and *Kras^LA2^/Mmp10^−/−^* mice. Columns, percentage of TBs; bars  = /−SEM, n≥50 TBs/genotype; p<0.001 Ntg vs. *Kras^LA^* mice; p<0.003 *Kras^LA^* mice vs. *Kras^LA2^/Mmp10^−/−^* mice; no significant difference in BASC number or distribution was observed between Ntg and *Kras^LA2^/Mmp10^−/−^* (p = 0.76) or Mmp10*^−^* ^/*−*^ (p = 0.76) mice.

The fact that *Mmp10*-deficient mice develop fewer *Kras*-mediated lung tumors suggests that *Mmp10* is involved in the initiating steps of *Kras*-mediated lung tumorigenesis *in vivo*. Initiation of *Kras*-mediated lung tumors is thought to involve clonal expansion of *Kras*-transformed bronchio-alveolar stem cells (BASCs), putative regional stem cells that reside at the terminal bronchioles adjacent to the alveolar space [29]. Therefore, we assessed whether *Mmp10*-deficiency affects the expansion of BASCs in response to urethane and oncogenic *Kras in vivo*. We identified and quantitated BASCs in paraffin sections of mouse lungs by dual immunofluorescence staining for surfactant protein C (SPC) and the Clara cell-specific protein (CCSP) as described previously [29], [30]. BASCs are observed at the bronchioalveolar duct junctions at terminal bronchioles as double SPC/CCSP positive cells (**Figure 3A**). Either urethane treatment or the presence of oncogenic *Kras* caused an expansion of BASCs at the terminal bronchioles (**Figure 3A**). Quantitative analysis of lung tissue sections from Ntg and *Mmp10^−/−^* mice in the absence or presence of urethane showed that urethane-treated Ntg mice exhibit a significant increase in the number of BASC per terminal bronchiole (BASCs/TB) when compared with control Ntg mice (**Figure 3B**). Urethane-treated *Mmp10^−/−^* mice showed a highly significantly diminiution in BASC expansion, such that the distribution and number of BASCs was not significantly different from untreated Ntg mice (**Figure 3B**). A similar inhibition of oncogenic BASC expansion was observed in *Kras^LA2^/Mmp10^−/−^* mice *in vivo* when compared to *Kras^LA2^* mice (**Figure 3C**). Therefore, in two independent models of *Kras*-mediated lung tumorigenesis, we observed that *Mmp10* deficiency leads to significant decreases in lung tumor number, size and burden; and in each model, the inhibitory effect of *Mmp10* deficiency on tumorigenesis was reflected in a defect in the oncogenic expansion of BASCs *in vivo*. Interestingly, Mmp10 deficiency does not appear to have an effect on BASC homeostasis per se since BASC number and distribution in Mmp10*^−^* ^/*−*^ mice is not significantly different from that of Ntg mice. Rather, Mmp10 appears to be important for oncogenic expansion of BASCs in response to urethane or *Kras* activation.

### Mmp10 is required for Kras-mediated BASC transformation in vitro

**Figure 4:**
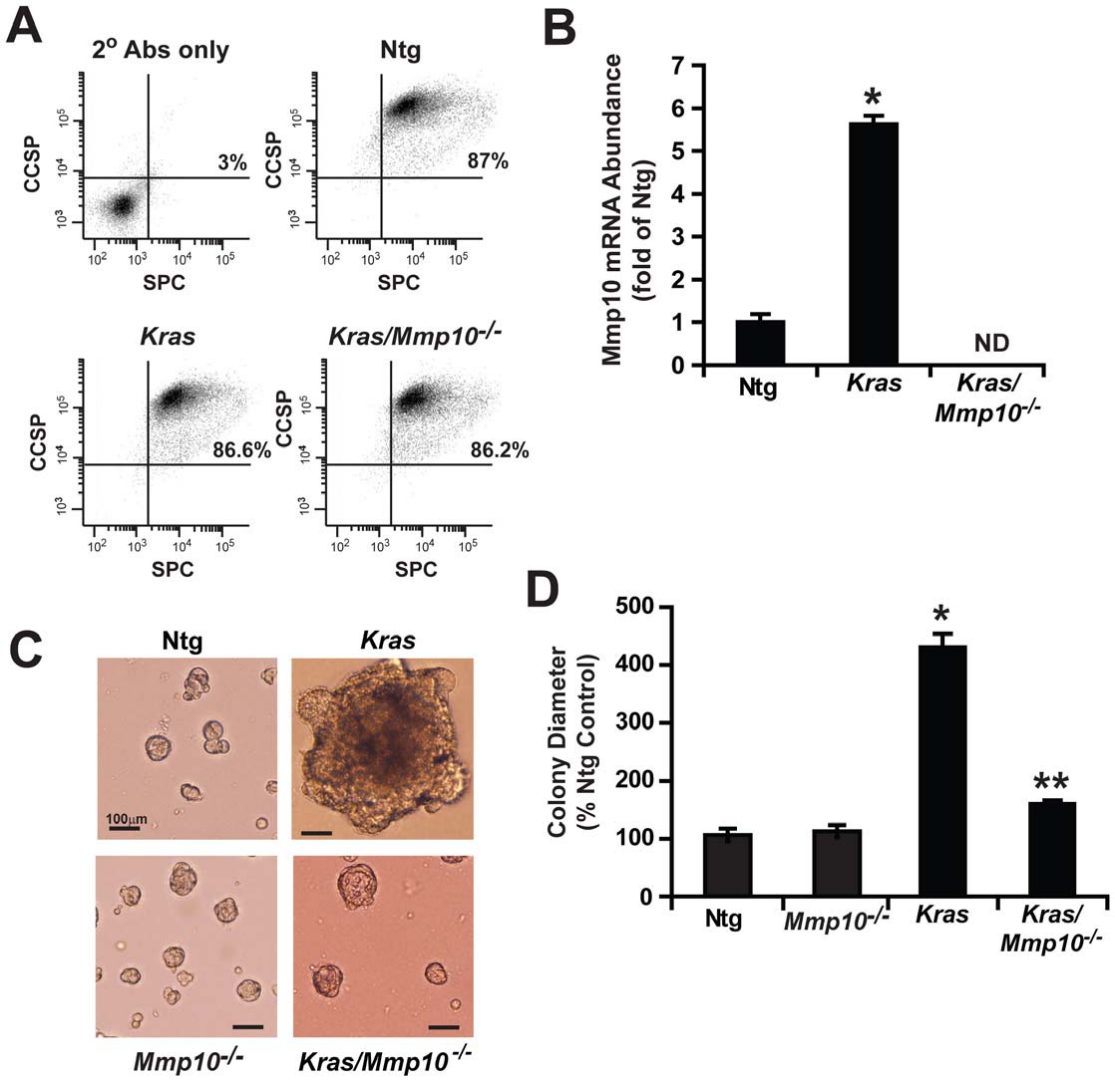
Mmp10 is required for *Kras*-induced expansion and transformation of BASCs *in vitro.* BASCs isolated from Ntg, *Mmp10^−/−^*, *LSL-Kras*, and *LSL-Kras/Mmp10^−/−^* mice were treated with AdCre and plated in three-dimensional Matrigel culture as described in *Experimental Procedures*. **A**) Flow cytometry of isolated BASCs for SPC and CCSP **B**) QPCR for Mmp10 in BASCs from Ntg, *LSL-Kras,* and *LSL-Kras/Mmp10^−/−^* mice. Fold of Ntg +/*−*SEM. n = 3, *p<0.000008. **C**) Morphology of BASC colonies from Ntg, *Mmp10^−/−^*,*LSL-Kras*, and *LSL-Kras/Mmp10^−/−^* mice. **D**) Analysis of BASC colony size. %Ntg +/−SEM. n = 85 Ntg, 56 *Mmp10^−/−^*,30 (*LSL-Kras*) and 80 (*LSL-Kras/Mmp10^−/−^*). *p<0.00001 Ntg vs, *LSL-Kras;* **p<0.00001 *LSL-Kras* vs. *LSL-Kras/Mmp10^−/−^*.

Given the importance of BASCs in tumor initiation, and the inhibitory effect of *Mmp10* loss on *Kras*-mediated BASC expansion *in vivo*, we assessed the role of Mmp10 in *Kras*-mediated BASC expansion and transformation *in vitro.* For this purpose, we utilized *LSL-Kras* mice, a model in which a conditional lox-stop-lox (LSL) *Kras* allele can be activated by Cre-mediated recombination [31]. BASCs were isolated from Ntg, *LSL-Kras,* and *LSL-Kras/Mmp-10^−/−^* mice and characterized for enrichment of BASCs using dual immunofluorescence and flow cytometry. Our BASC preparations consisted of >86% SPC/CCSP double positive cells when analyzed by flow cytometry confirming isolation of a highly enriched BASC population (**Figure 4A**). Treatment of BASCs from *LSL-Kras* mice with adenovirus expressing Cre-recombinase (AdCre) to activate the oncogenic *Kras* allele led to a significant increase in Mmp10 mRNA abundance, whereas AdCre-treated BASCs from *LSL-Kras/Mmp10^−/−^* mice expressed no detectable Mmp10 mRNA as expected (**Figure 4B**). These results confirm our previous finding that Mmp10 expression is induced in BASCs after expression of oncogenic *Kras* [30]. AdCre treated BASCs from Ntg or *Mmp10^−/−^* mice form small, highly organized spherical colonies when plated in three dimensional Matrigel culture of similar size and number (**Figure 4C**). In contrast, AdCre treated BASCs from *LSL-Kras* mice grow as larger amorphic, disorganized colonies characteristic of *Kras*-mediated transformation (**Figure 4C** **, middle panel;** [30]). AdCre treated BASCs from *LSL-Kras/Mmp10^−/−^* mice form colonies similar in size, number and morphology to those from Ntg and Mmp10*^−^* ^/*−*^ mice (**Figure 4C**). Quantitative analysis confirmed that BASC colonies from *LSL-Kras* mice are of larger diameter than colonies from Ntg, Mmp10*^−^* ^/*−*^ or *LSL-Kras/ Mmp10^−/−^* BASCs (**Figure 4D**). Thus, *Mmp10* is required for oncogenic *Kras*-induced morphological transformation and expansion of BASCs *in vitro* but has no appreciable effect on the maintenance of non-transformed BASCs in culture.

### Mmp10 expression is associated with stem cell signatures and metastasis in human lung cancer

**Figure 5:**
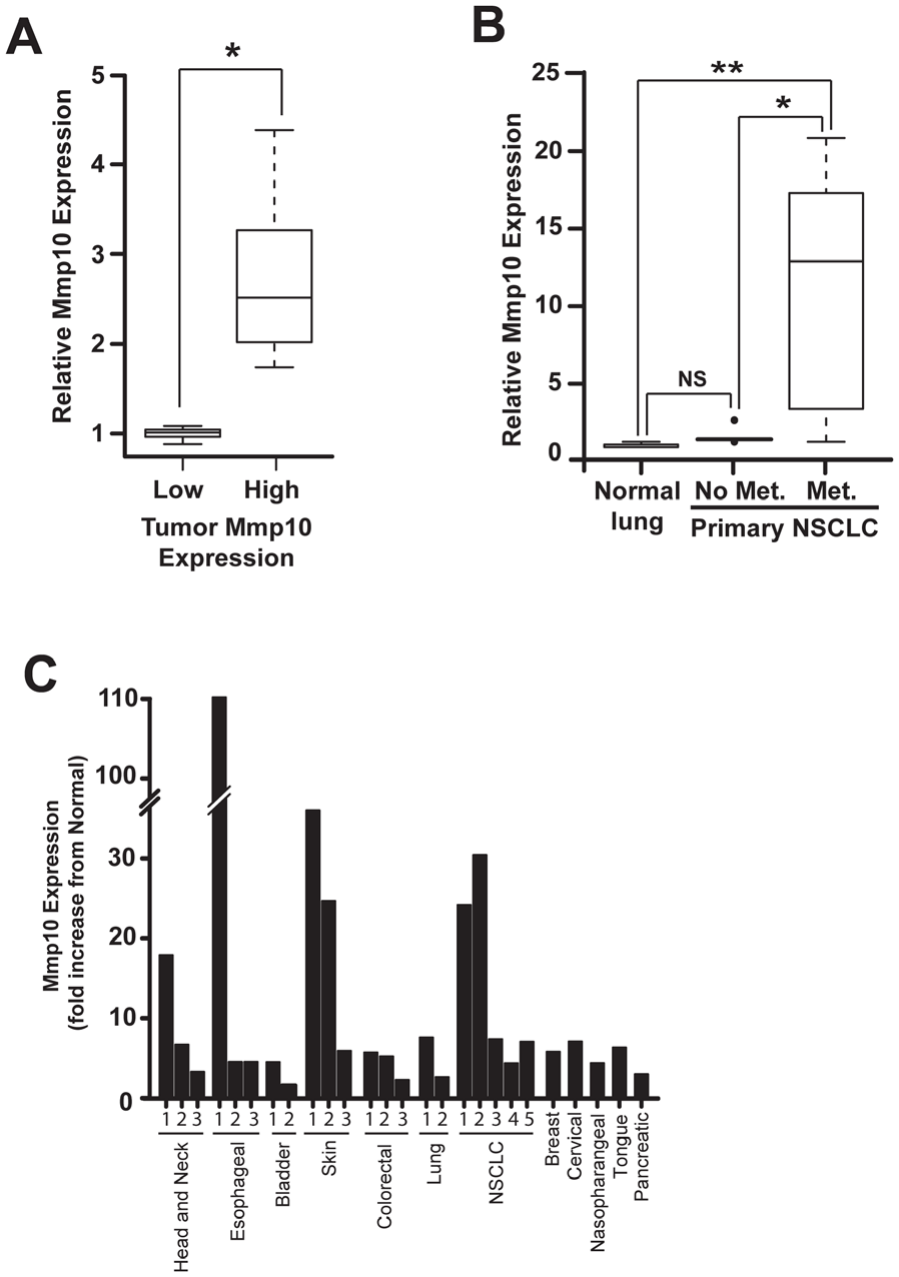
Mmp10 expression correlates with cancer stem cell genotypes and metastasis in human lung tumors. **A**) Gene expression data from primary human lung adenocarcinomas were divided into two groups of 30 samples consisting of lowest (Low) and highest (High) Mmp10 expressing lung tumors. n = 30; * p = 2.8×10^−33^. **B**) Mmp10 in normal lung versus lung tumors with (***Met.***) and without (***No Met.***) bone metastases. n = 3, normals, n = 9, No Mets, n = 7, Met.; NS =  not significant, *p = 0.008; **p = 0.04. **C**) Mmp10 mRNA expression in human tumors. Data are expressed as fold-change from matched normal.

We previously demonstrated that Mmp10 is overexpressed in human NSCLC and is important for transformed growth and invasion of human NSCLC cells *in vitro* [22]. Given the unexpected role of Mmp10 in expansion of *Kras*-transformed mouse lung BASC and tumor initiating activity, we computationally explored the relationship between Mmp10 expression, cancer stem cell expression profiles and metastasis in human lung cancers. For this purpose, we divided a publicly-available dataset consisting of genome-wide expression analysis of human lung adenocarcinomas (GSE11969) into two equal sized groups of 30 samples comprised of the tumors with the highest (High) and lowest (Low) Mmp10 RNA expression, respectively. Statistical analysis confirmed these two groups of samples express significantly different levels of Mmp10 mRNA (**Figure 5A**). We then performed gene set enrichment analysis (GSEA) against gene sets available as part of the Molecular Signatures Database (MSig) Version 3.0 as described in *Materials and Methods* to measure any association between Mmp10 and cancer stem cell gene expression profiles. Gene sets were identified by searching the MSig database for gene signatures containing the terms “cancer” and “stem” within their descriptions. GSEA revealed that 37 of the 50 (74%) cancer stem cell signatures were enriched in the high Mmp10 samples, and that 14 signatures were significantly enriched with a p-value <0.05 and FDR <25% (**Table 1**). Interestingly, only 1/50 stem cell signature was enriched in the low Mmp10 samples. This signature described genes that are downregulated in glioma stem cells, [32], further supporting the association of high Mmp10 with the cancer stem cell genotype. A separate GSEA using an independent lung adenocarcinoma gene expression data set (GSE13213) validated our initial analysis. 44/50 stem cell signatures were enriched in high Mmp10 tumor samples and none were enriched in the low Mmp10 tumor samples from this second data set. 10 stem cell gene sets were significantly enriched with a p-value <0.05 (**Table 2**), of which seven were also significantly enriched in the first lung adenocarcinoma gene set. These data provide compelling evidence that high Mmp10 expression in human NSCLC tumors is associated with a cancer stem-like gene expression profile.

**Table 1:** Cancer Stem Cell Signatures correlate with high Mmp10 expression in lung cancer.

Signature Name/Description	Size (genes)	ES	NES	P-value	FDR	Reference
Embryonic Stem Cell (ESC)-proliferation	111	0.44	2.11	<0.0001	0.003	[54]
ESC-Core signature	268	0.34	1.86	<0.0001	0.015	[55]
ESC-gene set 1	285	0.3	1.65	<0.0001	0.059	[54]
ESC-NOS targets	136	0.3	1.48	0.014	0.156	[54]
ESC-PRC2 targets	489	0.24	1.4	0.002	0.23	[54]
ESC-SUZ12 targets	765	0.23	1.38	<0.0001	0.23	[54]
ESC-Oct4 targets	228	0.25	1.35	0.023	0.21	[54]
ESC- EED targets	778	0.21	1.29	0.002	0.21	[54]
ESC-cycling genes	460	0.22	1.25	0.027	0.24	[54]
Breast cancer progenitor cells	266	0.24	1.32	0.021	0.22	[56]
ESC-H3K27 ME3	830	0.21	1.29	0.002	0.23	[54]
ESC-Myc-Max targets	653	0.21	1.24	0.015	0.23	[54]
ESC-Sox2 targets	509	0.21	1.22	0.018	0.22	[54]
ESC-Nanog targets	694	0.2	1.17	0.036	0.25	[54]

**Table 2:** Validation of the association between high Mmp10 in lung tumors and cancer stem cell signatures.

Gene Set	Size (genes)	ES	NES	p-value	Reference
**ESC-Proliferation**	143	0.5	2.14	<0.0001	[54]
**Breast cancer progenitor cells UP**	370	0.38	1.8	<0.0001	[56]
Breast cancer-tamoxiphen resistance	49	0.49	1.72	0.025	[57]
UV response cluster G4	13	0.65	1.72	0.044	[58]
**ESC-ES geneset 1**	370	0.33	1.56	0.004	[54]
Leukemic stem cells	234	0.34	1.53	0.007	[59]
**ESC-EED Targets**	1002	0.26	1.36	0.009	[54]
**ESC-cycling genes**	630	0.26	1.32	0.023	[54]
**ESC-H3K27ME3**	1058	0.24	1.25	0.018	[54]
**ESC-SUZ12_Targets**	979	0.24	1.25	0.037	[54]

Gene sets marked in bold text were also significantly enriched in the high Mmp10 lung tumor gene set analysis outlined in Figure 5 and Table 1.

We also performed an unbiased analysis using every gene set in the MSig database. In this analysis, the top stem cell signatures remained significantly enriched (*data not shown*). **Table 3** shows the most highly significant gene signatures associated with high Mmp10. Significantly, 3 of the top 5 gene sets identified contain Mmp10 as part of the signature. The top scoring gene set describes a signature comprised of genes over-expressed in early stage head and neck tumors suggesting an involvement in early tumorigenesis [33]. Among the gene sets that significantly correlate with high Mmp10 expression, many related to tumor progression, poor clinical outcome and metastatic potential. To specifically explore the association between Mmp10 and lung tumor metastasis, we analyzed a third gene expression dataset from early stage human lung adenocarcinoma samples that had produced a metastasis in bone tissue compared to samples that had not [34]. Analysis revealed that Mmp10 expression was significantly elevated in the primary tumors that produced metastases when compared to normal lung tissue but not in those that did not, indicating an association between Mmp10 expression and metastatic potential (**Figure 5B**). Interestingly, GSEA of the metastatic lung cancer gene set demonstrated a highly significant correlation with 10 stem cell signatures (p-value <0.05 and FDR <25%), four of which were also among the most significant gene sets identified using GSEA of the high Mmp10 lung adenocarcinomas (**Table 4**). These latter results reveal an association between metastatic potential and stem-like characteristics in primary human lung adenocarcinomas.

**Table 3:** The 10 most highly correlated signatures associated with high Mmp10 in lung cancer.

Gene Set Name	Size (genes)	ES	NES	p-value	FDR	Reference
**Upregulated in early stage Head and Neck tumors vs. normal**	43	0.75	2.65	<0.0001	<0.0001	[33]
**Up regulated in colon adenomas vs. normal mucosa**	128	0.6	2.51	<0.0001	0.001	[60]
Upregulated in breast cancer	19	0.85	2.41	<0.0001	0.009	[61]
**Up regulated in basal mammary epithelial cells vs. luminal**	53	0.67	2.4	<0.0001	0.007	[62]
Upregulated in cervical cancers and associated with proliferation and pooor outcome	140	0.56	2.38	<0.0001	0.011	[63]
Upregulated in NSCLC and predict poor survival	445	0.48	2.37	<0.0001	0.012	[64]
Up regulated in in advanced vs early gastric cancers	162	0.55	2.35	<0.0001	0.016	[65]
Up regulated in nasopharyngeal carcinoma vs. normal	272	0.5	2.31	<0.0001	0.026	[66]
Up regulated in invasive ductal breast carcinoma vs. carcinoma in situ	343	0.49	2.31	<0.0001	0.024	[67]
Up regulated in breast ductal carcinoma vs. normal	66	0.6	2.3	<0.0001	0.024	[68]

Gene sets marked in bold text contain Mmp10 as part of the gene signature.

**Table 4:** Association of metastatic lung cancer genes with cancer stem cell signatures.

Gene Set	Size (genes)	ES	NES	p-value	FDR	Reference
UV Response Cluster G4	14	0.67	1.86	0.005	0.015	[58]
Response to UV NHEK UP	146	0.41	1.82	<0.0001	0.012	[58]
UV Response Cluster G3	11	0.66	1.68	0.011	0.043	[58]
Silenced by Methylation in Colon Cancer	42	0.44	1.58	0.015	0.08	[69]
TP63 Gamma Targets	9	0.65	1.57	0.035	0.069	[70]
**ESC-EED Targets**	921	0.29	1.51	<0.0001	0.107	[54]
Cancer Progenitors	61	0.37	1.45	0.025	0.129	[54]
**ESC-PRC2 Targets**	566	0.28	1.44	<0.0001	0.124	[54]
**ESC with H3K27ME3**	991	0.27	1.44	<0.0001	0.111	[54]
**ESC SUZ12 Targets**	906	0.25	1.33	<0.0001	0.222	[54]

Gene sets marked in bold text are cancer stem cell signatures also identified as highly correlated with lung tumors expressing high Mmp10.

Given the importance of elevated Mmp10 expression in human lung cancer biology, we next assessed whether Mmp10 expression was also elevated in other forms of human cancer. Gene expression profiling revealed that Mmp10 is commonly overexpressed in many forms of human cancer, including lung, head and neck, esophageal, bladder, skin, colorectal, breast, cervical, nasopharyngeal, tongue and pancreatic cancers (**Figure 5C** and **Table 5**), suggesting a widespread role for Mmp10 in human malignancy.

**Table 5:** MMP10 is Overexpressed in many Human Cancer Types.

Tumor Type/Data set	Description	Tumor N	Control N	MMP10 T/N	p-value	Reference
Esophageal Cancer	Esophageal Tumor vs. matched normal	13	13	4.56	0.0158	[71]
Breast Cancer	Infiltrating ductal mammary carcinoma vs. normal	68	61	5.79	1.4E-15	[72]
Bladder Cancer	Muscle Invasive Carcinoma vs. normal	13	9	4.54	0.0015	[73]
Bladder Cancer	Primary Resected Bladder Tumor vs. normal	165	10	1.59	0.0006	[74]
*Skin Cancer	Squamous cell carcinoma vs. normal	11	4	36	4.1E-07	[75]
*Skin Cancer	Basal cell carcinoma vs. normal	15	4	24.7	4E-06	[75]
Colorectal Cancer	Colon biopsies from colorectal carcinoma patients	15	8	5.7	0.0008	[76]
Colorectal Cancer	Colorectal Adenoma vs. normal	32	32	5.2	5.4E-10	[60]
Lung Cancer	Squamous cell carcinoma vs. normal	16	7	7.59	0.0002	[77]
Lung Cancer	Adenocarcinoma vs. normal	7	2	2.64	0.034	[77]
Cervical Cancer	HPV-positive cervical cancer vs. normal	20	8	7.09	8.4E-05	[78]
Colorectal Cancer	metastatic-versus non-metastatic	77	N/A	2.29	0.04	[79]
Skin Cancer	Melanoma vs. normal	14	4	5.9	0.0002	[80]
NSCLC	stage T2 vs T1	41	15	24.2	0.007	[80]
NSCLC	stage T4 vs T1	14	4	30.4	0.005	[80]
NSCLC	N2 vs N0	9	13	7.38	0.027	[80]
NSCLC	M1 vs M0	17	24	4.4	0.025	[80]
Nasopharyngeal Cancer	Nasopharyngeal Cancer biopsies vs. normal	25	3	4.41	2.10E-07	[81]
SCC of tongue	SCC tongue vs. normal	26	12	6.33	1.30E-06	[82]
esophageal SCC	vs matched normal	53	53	4.57	9.00E-10	[83]
NSCLC	squamous cell (18) vs. adenocarcinoma (40)	18	40	7.06	1.00E-04	[84]
Pancreatic cancer	PDAC vs matched normal	36	36	3.01	9.70E-05	[85]

## Discussion

Increasing evidence suggests that many cancers, including lung cancer, possess a small subpopulation of cells that exhibit hallmark traits of stem cells. These “cancer stem cells” are thought to be responsible for the initiation, maintenance, progression and metastatic spread of tumors. Most current treatment modalities for lung cancer ultimately fail, perhaps due to intrinsic resistance of CSCs to therapy, resulting in disease recurrence and decreased patient survival. Therefore, molecular characterization of the mechanisms that govern the survival and growth of CSCs may hold a vital key to developing more effective therapeutic strategies that will improve the clinical outcome of patients with lung cancer.

The matrix metalloproteinases (MMPs) have long been implicated in tumor progression and metastasis. We recently demonstrated that Mmp10 is overexpressed in NSCLC and is a critical target of oncogenic *Kras* required for transformed growth and invasion of human NSCLC cells *in vitro* [22]. Our current study provides compelling evidence that Mmp10 exerts its pro-tumorigenic effects, at least in part, by maintaining a population of CSCs that drive tumor initiation and metastasis. Not only is Mmp10 elevated in tumors developed in two different mouse models of *Kras*-induced lung adenocarcinoma, genetic knock out of *Mmp10* leads to formation of significantly fewer tumors, suggesting an effect of Mmp10 on tumor initiation. Consistent with this conclusion, Mmp10 abundance is highly elevated in BASCs transformed with oncogenic *Kras* [30], and genetic loss of *Mmp10* leads to a failure of BASCs expressing oncogenic *Kras* to expand *in vivo,* and undergo morphological transformation *in vitro*. Though it is still unclear whether BASCs represent regional lung stem cells, strong circumstantial evidence indicates that they are involved in tumor initiation in the mouse lung. BASCs undergo expansion and transformation in response to *Kras* activation [29], and genetic and/or pharmacological disruption of multiple key oncogenic pathway genes involved in *Kras*-mediated tumorigenesis, including *Prkci* [30], *Pik3ca* [35], and *Bmi1* [36], lead to inhibition of BASC expansion and *Kras*-mediated tumor formation *in vivo*. On the other hand, recent studies have demonstrated that both Sca1^+^ (including BASCs) and Sca1^-^ cells exhibit tumor-initiating activity in *Kras* mice, demonstrating that BASCs are not the sole source of tumor-initiating cells in this model [37]. These findings indicate that tumor genotype is an important determinant of tumor-initiating cells. In humans, lung adenocarcinomas, which frequently harbor *Kras* mutations, often develop at the bronchio-alveolar duct junction and display either airway or alveolar differentiation, or both [38], suggesting that some of these tumors may have originated from BASC-like cells.

Our data provide evidence that the role of *Mmp10* in lung CSCs is cell autonomous. Both the *Kras^LA2^* and urethane tumor models show Mmp10 staining in tumor cells, with little to no staining in tumor associated-stroma or morphological normal lung epithelium. More importantly, the tumor inhibitory effects of the genetic loss of Mmp10 are reflected in a defect in oncogenic expansion of BASCs *in vivo* and *in vitro*. Thus, while many MMPs produced by the tumor microenvironment play prominent roles in the invasive and metastatic properties of lung tumor cells, our data demonstrate that Mmp10 specifically functions to support the autonomous growth of CSCs. However, our data do not exclude a contributory role for Mmp10 produced by and/or exerting its effects upon the tumor microenvironment. However, our studies do provide new insight into a largely unappreciated role for Mmp10 in the regulation of CSC behavior. Interestingly, Mmps have been implicated in regulation of tumor cell growth through cleavage and activation of cell surface proteins involved in cell growth regulation such as Notch [39], [40], and through proteolytic liberation of active growth factors such as TGFβ, IGF and TNFα from latent extracellular stores [41]. Future studies will focus on determining the specific molecular mechanisms that contribute to Mmp10-mediated CSC proliferation.

Cancer stem cells (CSCs) are defined by their ability to clonally expand, initiate tumors, maintain tumor progression and participate in tumor metastasis. The phenotype of these cells is associated with a genotype related to that of embryonic stem cells. Our finding that Mmp10 expression is associated with CSC genotypes in human lung tumors provides compelling circumstantial evidence that Mmp10 plays a critical role in maintenance of CSCs within human lung tumors. In this regard, it is interesting to note that Mmp10 expression has been observed to be elevated in tumor-initiating stem-like cells isolated from human small cell lung cancer cell lines [42], suggesting that Mmp10 may also function in the maintenance of these CSCs.

The leading cause of cancer-related deaths in lung cancer patients is metastatic dissemination. CSCs are thought to be the cells within a tumor that have the capability of metastasizing to distant sites. Our finding that Mmp10 is highly expressed in lung cancer-initiating BASCs, and is associated with the CSC genotype in human lung tumors suggests that Mmp10 may promote both CSC maintenance and metastatic potential through its role in CSC proliferation and metastatic behavior. Our finding that Mmp10 is elevated in human CSCs and that Mmp10 is highly expressed at the interface between mouse lung tumors and the surrounding tissue, suggesting a role for Mmp10 in tumor invasion, and are consistent with our previous funding that Mmp10 is required for invasion of human NSCLC cells *in vitro* [22]. These areas of increased Mmp10 staining may represent resident CSCs. The fact that Mmp10 is more highly expressed in tumors with high metastatic potential, and in the metastatic lesions of these tumors is consistent with the proposed role of CSC in metastatic spread. However, we cannot formally rule out an additional role for Mmp10 in bulk tumor cells that contributes to the metastatic potential of tumors. Our expression profiling data of human tumors demonstrates a close functional link between CSC, Mmp10 expression and metastatic potential, suggesting that Mmp10 plays a similar role in human lung adenocarcinoma CSC invasion and metastasis. Our results also demonstrate that MMP10 is highly expressed in many human tumor types, and is associated with poor outcome, metastatic potential and cancer stem cell signatures. These findings suggest a widespread role for Mmp10 in human malignancy and identify Mmp10 as a novel therapeutic target in cancer stem cells.

## Materials and Methods

### Mouse Urethane- and Kras^LA2^-mediated Lung Tumorigenesis Studies

Nullizygous *Mmp10* (*Mmp10^−/−^*) mice were obtained from the National Cancer Institute Mutant Mouse Regional Resource Center (MMRRC). The mice were generated on a mixed 129/C57BL/6J background and harbor a targeted disruption of exons 1–3 of the mouse *Mmp10* gene. The mice were bred onto a pure C57BL/6J background through 10 generations. Genotyping was conducted by PCR using primers recommended by MMRRC. *Mmp10^−/−^* mice and non-transgenic littermates were injected intraperitoneally with urethane at 1 mg/kg body weight weekly for six weeks to induce lung tumors. Control mice were injected with saline. Mice were analyzed twelve weeks after the first injection for the presence of pulmonary lesions. *Kras^LA2^* mice, generated as previously described [27], were mated with *Mmp10^−/−^* mice to generate bitransgenic *Kras^LA2^/Mmp10^−/−^* mice. *Kras^LA2^* and *Kras^LA2^/Mmp10^−/−^* mice were harvested at the time points indicated to assessed tumor number, tumor size, tumor burden and pathological classification by a board-certified pathologist (A.K.). *Mmp10^−/−^* and non-transgenic mice served as negative controls. All animal experiments were approved by the Institutional Animal Care and Use Committee of Mayo Clinic and were conducted under approved IACUC protocol# A30308. Mouse tissues were prepared for histology and immunohistochemistry as previously described [30], [43]. Sections were stained for Mmp10 (NBP1-03118; Novus Biologicals, Littleton, CO) and antigen visualized using the Envision Plus Dual Labeled Polymer Kit (DAKO). Images were analyzed using the ScanScope scanner and ImageScope software (Aperio Technologies, Vista, CA).

### BASC detection, isolation and culture ex vivo

BASCs were quantified in formalin fixed, paraffin embedded mouse lung tissues as described previously [30], [35]. *LSL-Kras* mice [44] were crossed with *Mmp10^−/−^* mice to generate bitransgenic *LSL-Kras/Mmp10^−/−^* mice. Lung epithelial cells were isolated from Ntg, *Mmp10^−/−^*, *LSL-Kras*, and *LSL-Kras/Mmp10^−/−^* mice and BASC isolation, Cre-recombinase treatment and *ex vivo* culture were carried out as described previously [30]. Brightfield images of BASC colonies were captured on an Olympus IX71 inverted microscope. BASC colony size was assessed using Image-Pro Plus 6.3 (Media Cybernetics, Bethesda, MD). BASCs were recovered from Matrigel culture for QPCR analysis using BD cell recovery solution (BD Biosciences).

### RNA isolation and quantitative PCR

Total RNA was extracted from BASC cells using the RNeasy Plus Mini Kit (Qiagen, Valencia, CA). QPCR reagents for mouse Mmp10 mRNA were purchased from Applied Biosystems (Foster City, CA). QPCR was carried out using an Applied Biosystems 7900 thermal cycler, and data was analyzed using the SDS 2.3 software package. Data were normalized to 18S RNA.

### Flow cytometry

BASCs were incubated for 1 hour at 4°C with Alexa Fluor 488-conjugated CD133 (Millipore, Billerica, MA) and Alexa Fluor 647 conjugated Notch4 (Biolegend, San Diego, CA) antibodies or respective isotype controls. Cells were incubated for 1 hour at 4°C with CCSP (Upstate, Temecula, CA) and SPC (Santa Cruz Biotechnology, Santa Cruz, CA) antibodies followed by a 30 minute incubation with Alexa Fluor 488- and Alexa Fluor 647 conjugated secondary antibodies (Invitrogen, Carlsbad, California). Flow cytometry was performed on an Accuri C6 flow cytometer and analyzed using CFlow Plus software (Accuri Cytometers, Inc., Ann Arbor, MI).

### Statistical analysis

Differences in the number and distribution of BASCs in the terminal bronchioles were assessed using the Cochrane-Armitage test using StatsDirect 2.6.1. Differences in tumor grade were assessed using the Mann-Whitney U test in Stats-Direct 2.6.1. The Student's *t* test and one-way ANOVA statistical analyses were done using SigmaStat 3.5. A *P* value of less than 0.05 was considered statistically significant.

### Assessment of Mmp10 in primary human cancer datasets

The correlation between Mmp10 and various types of human cancer was determined using the NextBio data mining framework (www.nextbio.com) [45]. The degree of correlation calculated by NextBio was based on Mmp10 values for individual microarray studies of specific cancer types. Selected gene expression experiments were chosen for a more in depth analysis The Gene Expression Omnibus (GEO) accession numbers for these studies were: GSE3292, GSE3292, GSE6631, GSE6059, GSE5364, GSE14999, GSE3167, GSE13507, GSE7553, GSE7553, GSE4183, GSE8671, GSE1987, GSE10799, GSE6791, GSE18105, GSE11117, GSE13597, GSE9844, GSE23400, GSE10939, and GSE15471. From these experiments, p-values and fold change measurements, provided by NextBio, were recorded.

### Gene Set Enrichment Analysis (GSEA) of Human Lung Cancer Data Sets

Three lung cancer gene expression datasets were analyzed to assess the relationships between Mmp10 levels, the cancer stem cell phenotype and metastasis in human cancer. The first two data sets (GSE11969 and GSE13213) are comprised of gene expression measurements from NSCLC tumors. [46], [47]. The third dataset (GSE10799) contained expression values from human lung adenocarcinoma samples that had produced metastasis in bone tissue compared to samples that had not [34]. All three of the microarray datasets were downloaded from GEO into the “R statistical computing language” using the “GEOquery” package of the “Bioconductor” software suite [48], [49], [50]. Quantile normalization of the datasets was performed using the “preprocess core” module [49], [51]. GSE11969 and GSE13213 were sorted according to their Mmp10 expression values. Lung tumor samples in GSE11969 were segregated into two sets. The first set contained the 30 samples with the highest Mmp10 expression values and the second set the 30 samples with the lowest Mmp10 expression values. GSE13213 was treated in the same manner except samples were separated into groups of 35 instead of 30. The size of the groups was determined to maximize the statistical significance of differential Mmp10 expression in each group as determined by a Welch's t-test.

Gene Set Enrichment Analysis (GSEA) was carried out on all of the lung cancer gene expression datasets described above [52], [53]. For each dataset, GSEA's were performed using two groups of gene sets that were available as part of the Molecular Signatures Database (MSig) Version 3.0 (http://www.broadinstitute.org/gsea/msigdb/index.jsp) [53]. The first collection of gene sets was intended to measure each datasets' degree of enrichment for the cancer stem cell phenotype. This group of gene sets was selected by searching the MSig database for signatures that contained the terms “cancer” and “stem” within their descriptions. The second collection of gene sets contained every signature listed in the MSig database and was intended to explore the relationships among the datasets in an untargeted fashion. In all GSEA's, gene sets that produced nominal p-values of less than 0.05 and false discovery rates (FDRs) of less than 0.25 were considered to be significantly enriched in the tested dataset.
